# Posterior monoaxial screw fixation combined with distraction-compression technology assisted endplate reduction for thoracolumbar burst fractures: a retrospective study

**DOI:** 10.1186/s12891-020-3038-6

**Published:** 2020-01-09

**Authors:** Xuhong Xue, Sheng Zhao

**Affiliations:** Department of Orthopedics, The Second Hospital of Shanxi Medical University, No. 382 Wuyi Road, Taiyuan, Shanxi 030001 People’s Republic of China

**Keywords:** Thoracolumbar injury, Monoaxial pedicle screws instrumentation, Burst fractures, Distraction-compression technology

## Abstract

**Background:**

The management of thoracolumbar burst fractures traditionally involves posterior pedicle screw fixation, but it has some drawbacks. The aim of this study is to evaluate the clinical and radiological outcomes of patients with thoracolumbar burst fractures. They were treated by a modified technique that monoaxial pedicle screws instrumentation and distraction-compression technology assisted end plate reduction.

**Methods:**

From March 2014 to February 2016, a retrospective study including 42 consecutive patients with thoracolumbar burst fractures was performed. The patients had undergone posterior reduction and instrumentation with monoaxial pedicle screws. The fractured vertebrae were also inserted screws as a push point. The distraction -compression technology was used as assisting end plate reduction. All patients were followed up at a minimum of 2 years. These parameters including segmental kyphosis, severity of fracture, neurological function, canal compromise and back pain were evaluated in preoperatively, postoperatively and at the final follow-up.

**Results:**

The average follow-up period was 28.9 ± 4.3 months (range, 24-39mo). No patients had postoperative implant failure at recent follow-up. The mean Cobb angle of the kyphosis was improved from 14.2°to 1.1° (correction rate 92.1%). At final follow-up there was 1.5% loss of correction. The mean preoperative wedge angle was improved from 17.1 ± 7.9°to 4.4 ± 3.7°(correction rate 74.3%). The mean anterior and posterior vertebral height also showed significant improvements postoperatively, which were maintained at the final follow-up(*P* < 0.05). The mean visual analogue scale (VAS) scores was 8 and 1.6 in preoperation and at the last follow-up, and there was significant difference (*p* < 0.05).

**Conclusion:**

Based on our experience, distraction-compression technology can assist reduction of collapsed endplate directly. Satisfactory fracture reduction and correction of segmental kyphosis can be achieved and maintained with the use of monoaxial pedicle screw fixation including the fractured vertebra. It may be a good treatment approach for thoracolumbar burst fractures.

## Background

Spinal fractures commonly occur in the thoracolumbar region, of which burst fractures accounting for 10–20% of all spinal fractures [1–3]. It was always attributed to biomechanical and anatomical features in thoracolumbar junction, which is the most important biomechanical transition zone between rigid thoracic kyphosis and flexible lumbar lordosis. Thoracolumbar burst fractures are serious injuries that can result in persisting back pain, kyphotic deformity and neurologic deficits [4]. Therefore, the treatment principles for these patients include spine stability restoration, kyphotic deformity correction and neural canal decompression as early as possible [5]. Currently, transpedicular screw instrumentation is the mainstream to treat thoracolumbar burst fractures. However, it was reported that short segment pedicle screw fixation was prone to failure in severe thoracolumbar fracture due to insufficient anterior column support [6, 7].

To prevent internal fixation failure, many efforts were done to reconstruct the anterior column including transpedicular bone grafting, vertebroplasty and direct anterior reconstruction [8]. However, direct anterior corpectomy and reconstruction was always confronted with higher complication rates and major injuries. Furthermore, reduction of injured vertebra in most cases is unsatisfactory, and patients are usually suffered from continuous backache. Therefore, adequate fracture reduction was very important for less implant failures and good clinical outcomes. How can we resolve these problems?

It was reported that restoration of the intervertebral disc space boundaries might prevent intrusion of the disc in the injured vertebra body after pedicle screw fixation [9]. Posterior fixation including the fractured vertebra, also called intermediate screws technology, has significantly biomechanical advantages over conventional short-segment fixation [10]. Herein, the posterior fixation including the fractured vertebra combined with distraction-compression technology assisted end plate reduction was performed in patients with thoracolumbar burst fracture. The purpose is to evaluate and analyze the radiological and clinical outcome of this technique.

## Methods

This study has been approved by institutional review board of our hospital. Inclusion criteria: (A) traumatic thoracolumbar burst fracture (T10-L2); (B) acute injury less than 3 weeks; (C) with or without s neurological impairment; (D) intact radiographic data in preoperatively, postoperatively and at the final follow-up. Exclusion criteria: (A) Other trauma in addition to spinal trauma; (B) Pathologic fracture; History of malignancy; (C) Previous neurological injuries; (D) pediatric (< 16 years of age) and non-operatively treated patients; (E) Comorbidities (diabetes, chronic kidney failure, steroid use). From March 2014 to February 2016, forty-two consecutive patients met the criteria. There were 36 male and 6 female, with an average age of 38.7 ± 10.9 years (range, 16–62 years). The injuries were caused by falls from a high place(23 patients), traffic accidents (16 patients), and being hit by falling object (3 patients). All patients underwent plain X-rays, computed tomography (CT) and magnetic resonance imaging (MRI) before surgery. Neurologic examination was record by spine surgeons at the time of admission. The neurological status was assessed using the American Spinal Injury Association impairment scale (ASIA). The back pain was evaluated using the VAS scale in pre-operation and at the final follow-up. All patients were evaluated using comprehensive AO classification and score of the thoracolumbar injury classification and severity (TLICS) for thoracolumbar fracture [11].

**Fig. 1:**
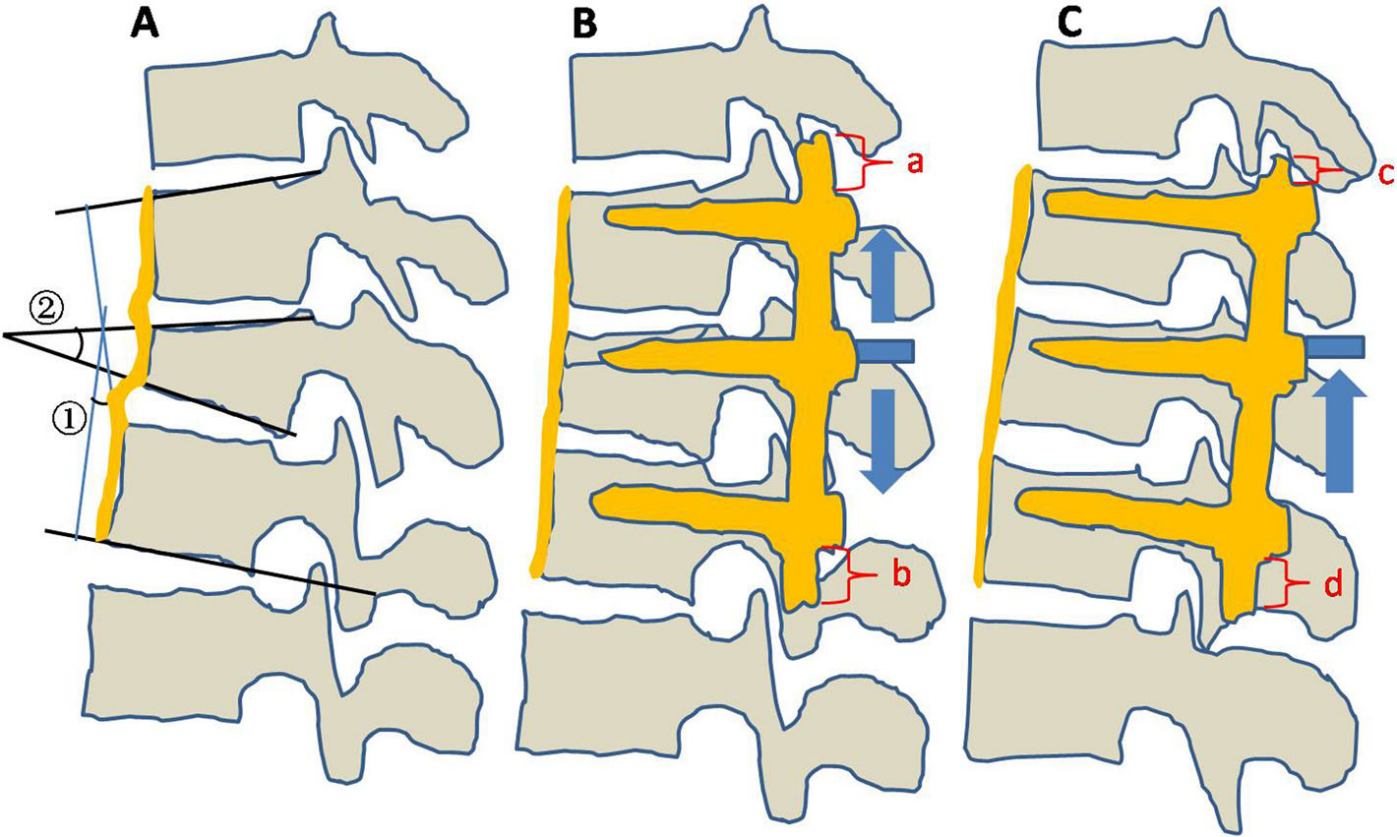
**a**. Radiological evaluation using plain lateral radiography. ①:Kyphosis angle, ②:vertebral wedge angle; The anterior longitudinal ligament is curly in fracture region. **b**. Correction of the segmental kyphotic angle was performed indirectly via connecting rods distraction proximally and distally in sequence. The ALL and PLL were straightened as the ligamentotaxis. **c**. Reduction of the endplate collapse was performed directly by longitudinal compression of intermediate screws

The radiological parameters include kyphotic angle, vertebral wedge angle (Fig. 1a), anterior and posterior edge height of injured vertebra. All patients had preoperative CT scans, which were studied and recognizable bony narrowing of the spinal canal. According to Mumford’s modality, the percentage of canal compromise was calculated with the mid-sagittal diameter of the injured level divided by the mean of the mid-saggital diameters of adjacent upper and lower levels [12].

### Technology

**Fig. 2:**
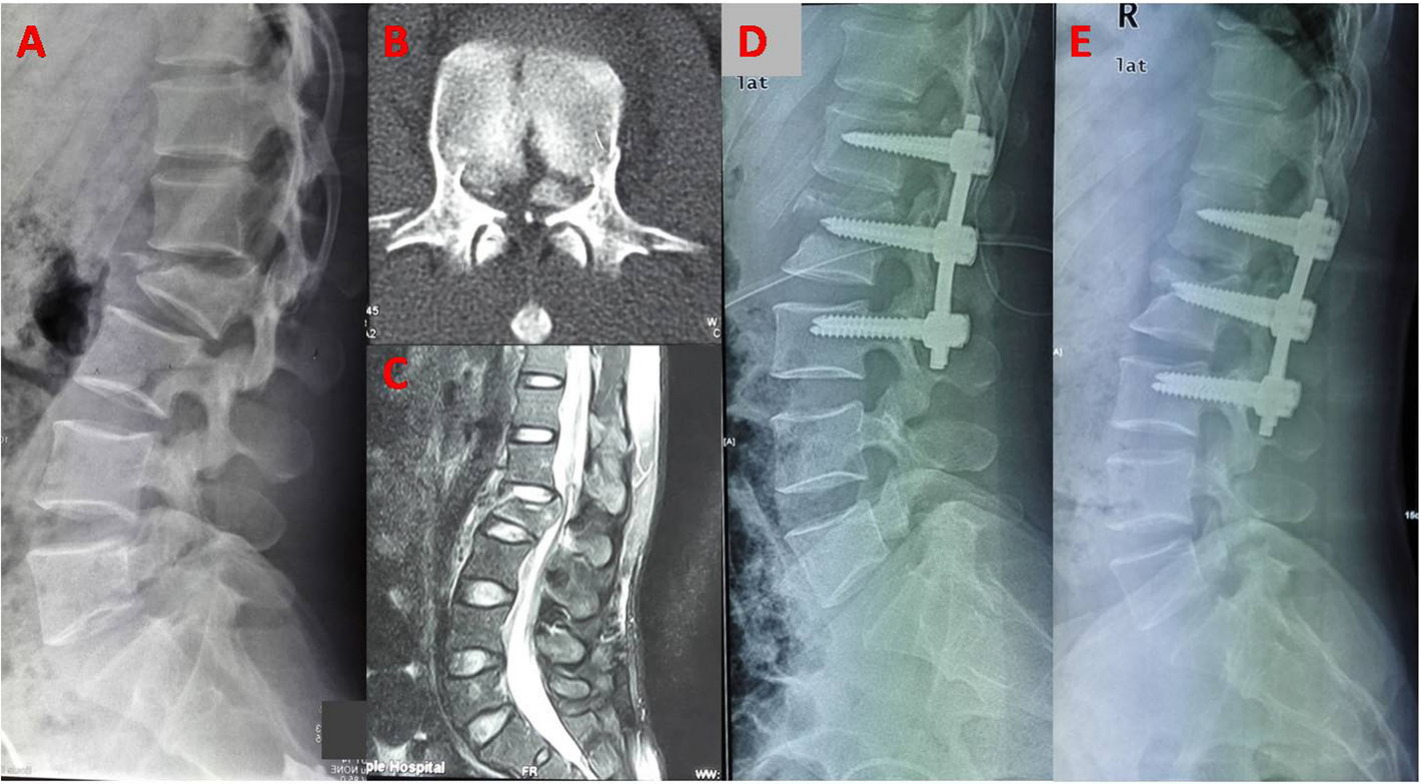
**a.** A 35-year-old man with an L2 burst fracture; TLICS score is 6 points. **b.** Axial computed tomography image shows comminution and canal encroachment. **c.** Sagittal MRI shows PLC injury. (D)Two-level posterior fixation was done from L1 to L3. **d.** X-ray shows minimal loss of correction at 27 months after surgery

All surgical procedures were performed under general anesthesia. Patients were positioned prone on the Jackson frame in order to reduce the intra-abdominal pressure. In addition, it could create a positional reduction effect on the fracture. We exposed the levels above and below the injured segment via posterior midline approach. Monoaxial pedicle screws were inserted to bilateral pedicles of the adjacent vertebrae. Then, intermediate screws were inserted into the pedicles of the fractured vertebrae parallel to the endplate of the adjacent normal vertebrae in a convergent direction. Reduction of fractured vertebrae, as well as collapsed endplate, was achieved via use of distraction-compression technology. Details of reduction procedure were as follows: firstly, intermediate screws were locked temporarily. Indirect reduction of collapsed vertebral bodies and correction of the segmental kyphosis were performed via ligamentotaxis after connecting rods distraction toward the cranially and caudally in sequence. The ALL and PLL as ligamentotaxis were straightened; the length of the spine was restored (Fig. 1b). Secondly, direct reduction of the collapsed endplate was performed by longitudinal compression of intermediate screws (Fig. 1c). As a result, satisfactory reduction of height loss and the correction of the kyphotic deformity were achieved (Fig. 2).

**Fig. 3:**
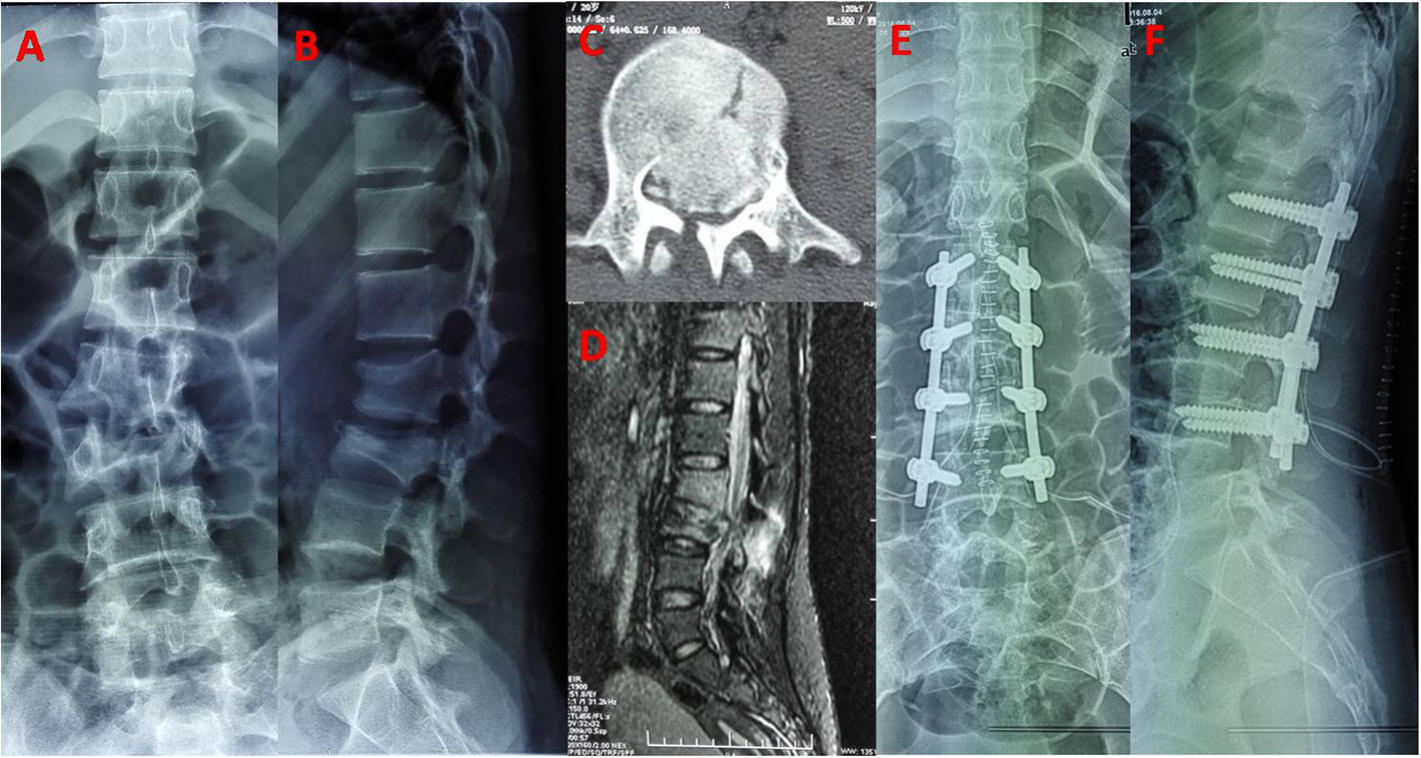
**a, b.** A 20-year-old woman with an L3 burst fracture and incomplete paralysis. **c.** Axial CT image shows comminution and canal encroachment. **d.** Sagittal MRI shows PLC injury. **e, f.** Posterior monoaxial pedicle screw fixation was done from L1 to L4. The kyphotic deformity was corrected and fractured vertebra height was restored

For patients without or mild nerve damage, if the bone fragment in posterior margin of vertebra is not reversed, laminectomy decompression was not performed even when the spinal canal stenosis was more than 50% of its normal value (Fig. 2). Spinal canal compromise can be improved after indirect reduction via ligamentotaxis [13]. However, for patients with moderate or above neurologic deficit (ASIA A,B,C), posterior decompression was performed by focal laminectomy (Fig. 3). For all 42 patients, procedures were checked by lateral-view radiographs. The rehabilitation program was started the day after surgery. Bracing was prescribed for patients during 6 weeks after surgery. All patients were followed up clinically and radiographically at 1 week, 1 month, 3 month, 6 month, 1 year and 2 years postoperatively. Anteroposterior and lateral X-ray were done in prone position in preoperative because of spine fracture, while it was done in standing position in postoperatively and at the follow up. Implants removal was performed at 1 to 2 years after surgery when bone healed. For patients with bony canal narrowing, CT scan was performed at 1 week and 2 years in postoperatively.

### Statistical analysis

Statistical analysis was performed using IBM SPSS ver.19.0 (IBM Co., Armonk, NY, USA). Compared t-tests were used for radiologic data analysis. The level of significance was set at *P* < 0.05 for all statistical tests.

## Results

### Clinical results

The level of spinal involvement was T11 in 4 patients, T12 in 17 patients, L1 in 12 patients, L2 in 6 patients, and L3 in 3 patients. Twenty-seven patients had normal neurologic function (ASIA E), 11 had incomplete neurologic deficits (ASIA B, C, D), and four had complete neurologic deficit (ASIA A). According to AO comprehensive classification system, 9 were Type A4, 6 were type A3, 7 were type A2, 12 were type B2, and 8 were type C. The mean score of the TLICS was 6.2 ± 2.0 points (range, 4–9 points). The average follow-up period was 28.9 ± 4.3 months (range, 24-39mo).

Posterior instrumented stabilization and decompression were performed in 7 patients with severe neurologic deficit (ASIA A,B,C). In eight patients, four segments were fixed because of coexistent fractures at adjacent levels. Three segments including injured and adjacent vertebra were fixed in other 34 patients. Pedicle screws were inserted into fractured vertebra bilaterally in 40 patients; other two patients had only unilateral screws insertion due to contralateral broken pedicles. The mean operating time was 90 ± 36 min; the mean blood loss was 234 ± 95 ml. Three patients with complete spinal cord injury (ASIA A) improved to ASIA B; another one ASIA A patients improved to ASIA D. Postoperative neurologic status of all patients were summarized in Table 1. Thirty-three patients were undergone implant removal in one year later. Residual nine patients had no implant failure at the final follow-up. All patients received only posterior surgery and anterior decompression or reconstruction surgery were not performed.

**Table 1:** Neurologic function (ASIA Impairment Scale)

	Postoperative neurologic status (no. of patients)				
Preoperative	A	B	C	D	E
A		3		1	
B			1	1	
C					1
D					8
E					27

Back pain was evaluated using VAS scale. The mean VAS was 8 and 1.6 in pre-operation and at the last follow-up, and there was statistically significant difference (*P* < 0.05).

### Radiological results

The mean kyphotic angle, vertebral wedge angle, percentage of anterior and posterior edge vertebra height and spinal canal compromise are presented in Table 2. All of the parameters were significantly improved after surgery (*P* < 0.05). The mean preoperative kyphotic angle was 14.2 ± 10.0 degree, which was corrected to 1.1 ± 7.8 after surgery. At the final follow-up, the mean loss of reduction was to 1.3 degree. The mean preoperative wedge angle was 17.1 ± 7.9 degree. This was improved to 4.8 ± 3.7 degree(*p* < 0.05). The mean anterior and posterior vertebral height also showed significant improvements postoperatively, which were maintained at the final follow-up. The mean preoperative canal narrowing is 36.0%, which was recovered 16.0 and 3.1% after surgery and at the last follow-up, respectively.

**Table 2:** Radiological parameters evaluated at the preoperatively, postoperatively, and final follow up

Radiological parameters	Pre-operation	Post-operation	Final follow up
Kyphotic angle (°)	14.20 ± 9.99	1.12 ± 7.80*	2.45 ± 5.50^△^
Vertebral wedge angle (°)	17.14 ± 7.87	4.14 ± 3.44*	4.37 ± 3.68^△^
Canal compromise (%)	36.02 ± 28.97	16.00 ± 10.09*	3.05 ± 3.67^△^
AH (%)	62.66 ± 23.48	92.36 ± 46.78*	91.15 ± 36.28^△^
PH (%)	92.03 ± 33.12	96.8 ± 40.36*	96.65 ± 35.40^△^

Notes: AH, anterior edge height of fractured vertebra; PH, posterior edge height of fractured vertebra. *P < 0.05, ^△^*P* > 0.05

## Discussion

Traditionally, the management of thoracolumbar burst fractures involves posterior pedicle screw fixation at the levels adjacent to the fractured vertebra. However, it was found that this method of stabilization has high failure rates, which manifested as progressive loss of fracture reduction, screw breakage and segmental kyphosis [14]. Aono H,et al. had suggested that the deficient anterior column was the main reason of failures after short-segment posterior fixation biomechanically [15]. In this situation, intermediate screws technology shows more advantage than prior short-segment posterior fixation. It can function as a push point with an anterior vector, which creates a lordorizing force so as to correct the segmental kyphosis. Meanwhile, the cantilever effects caused kyphosis were decreased by the three-point fixation [16, 17].

In present study, adequate reduction was achieved immediately after surgery. Firstly, partial reduction was obtained by the lordotic prone position. Secondly, kyphotic deformity was corrected by rod distraction and monoaxial pedicle screws, which were inserted parallel to the end plate of the adjacent vertebral body. Lastly, we tried to get endplate reduction through distraction-compression technology.

The success of this surgical procedure depends on these aspects as follows. Firstly, the use of intermediate screws can increase the fixation strength and stability of a posterior short segment construct. Secondly, the use of monoaxial pedicle screw can exhibit more correcting force for segmental kyphosis. A study has reported by Wang that monoaxial pedicle screw exhibited more stability in flexion and extension than the polyaxial pedicle screw [18]. Thirdly, the preservation of the posterior column is beneficial to reducing the stress concentration when anterior and middle columns injured; it may prevent early implants failure.

There are theoretical concerns regarding the use of intermediate screws. On the one hand, the pullout strength of the screw, no doubt, is weakening in the fractured vertebra body. However, the support and lever force were more needed for intermediate screws. On the other hand, whether is it safe to insert a screw through a broken bone, such as breach into the spinal canal? In fact, we found the walls of the pedicle are intact in most cases with burst fractures. The CT scan images have also demonstrated good containment of screws in postoperatively. So inserting screw in injured vertebrae is similar to pedicle screw insertion in adjacent vertebrae.

We did not perform laminectomy, even when patients had mild neurological deficits. Previous study by Shuman concluded there is no correlation between decompression and subsequent neurologic improvement [19]. Zhang et al. have also reported good neurological improvement was achieved in 36 patients with thoracolumbar fracture and incomplete neurological deficits. They were treated using posterior indirect reduction and pedicle screw fixation without laminectomy or laminotomy [20]. Boerger et al. concluded that geometrical parameters of canal compromise do not relate to initial neurological deficits [21]. On the contrary, Meves R et al. reported that narrowing of the spinal canal has a stronger association with neurologic deficit than injury severity in thoracolumbar burst fractures [22]. There are several studies showed a positive effect of decompression. These patients might have neurologic improvement if decompression was performed during the first 24 h after accident [23–25]. Nevertheless, the role and timing of surgery after spinal cord injury remain controversial. Stabilization of the spine, decompression of the spinal cord, and maintenance of blood perfusion are critical in optimizing neurological recovery. In present study, for the patients with moderate or above neurologic deficit, posterior instrumented and focal decompression were performed. All of patients showed neurological improvements in different degrees at the final follow up.

For canal narrowing, the radiological data in our study are similar to the past published case series. Spontaneous resorption of bone fragments in spinal canal has been reported in some cases with or without instrumentation [26, 27]. In present study, significant remodeling of the spinal canal was observed at one year after surgery. At the final follow up, bony narrowing of the spinal canal is only recognizable in 3.05%, which is in line with Leferink VJ et al. [28]. Because of comminuted bony fragments affect spontaneous canal remodeling, the change of resorption can be estimated by CT scan.

For the option of additional posterior fusion, there is still hot topic. In the past and present, fusion surgery has been regarded as the gold standard for spinal disorder. Theoretically, it is very likely that the rods will break during the first five years after stabilization if without fusion. However, a study by Jindal N et al. concluded that adjunctive fusion is unnecessary for thoracolumbar burst fracture with short segment pedicle screw fixation [29]. The advantages of non-fusion are preservation of mobility, shorten operating time, lesser blood loss and obviated donor site complications [30]. However, the disadvantage of non-fusion is the need for further surgery to remove the implants. In present study, transpedicular fixation without fusion was selected and implants removal was suggested in one year later.

There is still controversy regarding the implant removal. A retrospective study by Chou et al. including 69 cases has suggested that implant removal may be unnecessary for patients with thoracolumbar burst fractures after instrumentation without fusion. They concluded that the radiological and functional outcomes of both implant removal and retention were similar. However, information should be provided beforehand regarding the possibility of screw breakage [31]. On the contrary, a case-control study by Jeon CH concluded that implants removal is beneficial because it alleviates pain and disability [32]. Furthermore, restoration of the segmental mobility may contribute to the functional improvement.

This study has some limitations. First, forty-two patients is a rather small group for such a clinical study. Second, the timing of the removal of the implant and non-fusion still remain as an open question. Third, the retrospective nature of the study and short follow up time were also a limitation; it merits further investigation with a more prospective and controlled design. In addition, this study does not contain results about life quality in the follow-up. Further clinical parameters and scores (such as ODI, SF 36, patient satisfaction, return to work) about life quality, especially spinal mobilization and mental health status, are needed to be evaluated in the future.

## Conclusion

Based on our experience, distraction-compression technology can assist reduction of collapsed endplate directly. Satisfactory fracture reduction and correction of segmental kyphosis can be achieved and maintained with the use of monoaxial pedicle screw fixation including the fractured vertebra. It may be a good treatment approach for thoracolumbar burst fractures.

## Data Availability

The datasets used and/or analyzed during the current study are available from the corresponding author upon reasonable request.

## Abbreviations

ASIA: American Spinal Injury Association
CT: Computed tomography
MRI: Magnetic resonance imaging
SPSS: Statistic Package for Social Science
TLICS: Thoracolumbar injury classification and severity
VAS: Visual analogue scale
